# Third generation cephalosporin use in a tertiary hospital in Port of Spain, Trinidad: need for an antibiotic policy

**DOI:** 10.1186/1471-2334-4-59

**Published:** 2004-12-15

**Authors:** Lexley M Pinto Pereira, Marjorie Phillips, Hema Ramlal, Karen Teemul, P Prabhakar

**Affiliations:** 1 Department of Paraclinical Sciences, Faculty of Medical Sciences, The University of the West Indies, St Augustine, Trinidad; 2 Pharmacy, General Hospital, Port of Spain, Trinidad; 3 The Caribbean Epidemiology Center, Port of Spain, Trinidad

## Abstract

**Background:**

Tertiary care hospitals are a potential source for development and spread of bacterial resistance being in the loop to receive outpatients and referrals from community nursing homes and hospitals. The liberal use of third-generation cephalosporins (3GCs) in these hospitals has been associated with the emergence of extended-spectrum beta- lactamases (ESBLs) presenting concerns for bacterial resistance in therapeutics. We studied the 3GC utilization in a tertiary care teaching hospital, in warded patients (medical, surgical, gynaecology, orthopedic) prescribed these drugs.

**Methods:**

Clinical data of patients (≥ 13 years) admitted to the General Hospital, Port of Spain (POSGH) from January to June 2000, and who had received 3GCs based on the Pharmacy records were studied. The Sanford Antibiotic Guide 2000, was used to determine appropriateness of therapy. The agency which procures drugs for the Ministry of Health supplied the cost of drugs.

**Results:**

The prevalence rate of use of 3GCs was 9.5 per 1000 admissions and was higher in surgical and gynecological admissions (21/1000) compared with medical and orthopedic (8 /1000) services (p < 0.05). Ceftriaxone was the most frequently used 3GC. Sixty-nine (36%) patients without clinical evidence of infection received 3Gcs and prescribing was based on therapeutic recommendations in 4% of patients. At least 62% of all prescriptions were inappropriate with significant associations for patients from gynaecology (p < 0.003), empirical prescribing (p < 0.48), patients with undetermined infection sites (p < 0.007), and for single drug use compared with multiple antibiotics (p < 0.001). Treatment was twice as costly when prescribing was inappropriate

**Conclusions:**

There is extensive inappropriate 3GC utilization in tertiary care in Trinidad. We recommend hospital laboratories undertake continuous surveillance of antibiotic resistance patterns so that appropriate changes in prescribing guidelines can be developed and implemented. Though guidelines for rational antibiotic use were developed they have not been re-visited or encouraged, suggesting urgent antibiotic review of the hospital formulary and instituting an infection control team. Monitoring antibiotic use with microbiology laboratory support can promote rational drug utilization, cut costs, halt inappropriate 3GC prescribing, and delay the emergence of resistant organisms. An ongoing antibiotic peer audit is suggested.

## Background

Appropriate use of antibiotics is central to limiting the development and the spread of resistant bacteria in hospitals and communities. Use of broad-spectrum antibiotics, in particular the 3GCs in nosocomial infections have been linked to the emergence of antibiotic resistance and increase in costs [1]. The hospital setting is particularly conducive to the development of antibiotic resistance as patients who are severely ill, immuno-compromised or have devices and/or implants in them are likely to receive frequent courses of empirical or prophylactic antibiotic therapy [2]. Furthermore, the absences of guidelines for antibiotic use, protocols for rational therapeutics and infection control committees have led to overuse and misuse of antimicrobials in different specialized units in hospitals.

The increasing resistance to 3GCs accompanied by an increasing cost burden has raised concerns about the detection, prevalence, and clinical implications of infections with *Escherichia coli *and *Klebsiella spp*. An important source of this resistance results from the production of extended-spectrum beta-lactamases (ESBLs) by bacteria. ESBLs are modified beta-lactamase enzymes mainly derived from the ubiquitous TEM1/2 and SHV-1 plasmid-mediated enzymes, which hydrolyse expanded spectrum cephalosporins to varying degrees. Many beta-lactamases result in resistance to 3GCs in Enterobacteriaceae. Genera such as Enterobacter, Citrobacter and Serratia posses chromosomal broad spectrum beta-lactamases which are normally repressed, and when induced result in resistance to 3GCs. Klebsiella and E. coli usually have the SHV- or Tem- type beta-lactamases, and key mutations in these result in true "ESBLs". ESBLs have received attention in the last decade because although penicillins, cephalosporins, or aztreonam appear to be susceptible in vitro, ESBL producing *E. coli *or *Klebsiella *spp. may demonstrate clinical resistance to these antibiotics leading to treatment failures. Liberal use of the 3GC antibiotics has resulted in the ESBLs conferring resistance among Enterobacter[3] and Enterobacteriacae worldwide [4-6] compromising their clinical use. Prior antibiotic use is an important risk factor for colonization and bacterial infection and though generally antibiotic use cannot always be correlated with emergent antibiotic resistance, studies have reported the association of resistant *K pneumoniae *and other *Enterobacteriaceae *and vancomycin-resistant enteroccocci with cephalosporin use [7-11]. Recent increases in multidrug resistant gram-negative bacilli, particularly ESBLs is of great concern. The association between emergent ESBL-mediated infections and 3GC use emphasizes the importance of better describing 3GC drug utilization to best optimize their use. Few data are available in this regard from developing countries. In the Caribbean 3GC resistance amongst the *Enterobacteriaceae *has been reported from Barbados [12], and extended spectrum beta-lactamase producing *Enterobacteriaceae *has recently been observed in tertiary care in Jamaica [13] and Trinidad [14]. From unpublished reports of drug procurements for the public sector, the third generation cephalosporins are widely used at the General Hospital Port of Spain, which is the largest tertiary health care institution in the country. In order to assess the appropriateness of prescribing 3GCs and to determine the direct cost of treatment, an audit of prescriptions of these agents was undertaken at the POSGH, between January to June 2000.

## Methods

### Setting

In Trinidad and Tobago, health care is delivered through the public sector, though several private facilities are also available. At the publicly-financed health care institutes patients undergo investigations and receive treatment without cost. There are 2 tertiary general hospitals in Trinidad, the General Hospitals at Port of Spain and San Fernando and several secondary and primary health care facilities where patients can access medical care. The POSGH is the country's major health care institute and receives referrals from all over the country including the sister isle of Tobago. This hospital is a 900-bed institute providing out-patient services and health care for warded patients for over one third of Trinidad and Tobago's population of 1.3 million. Medical students undergoing training at the University of the West Indies, also attend clinical rotations at this hospital.

### Patients

Between January to June 2000, we conducted a cross sectional study of adult in- patients (over 13 years) who had received one or more courses of treatment with one of the 3GCs (cefotaxime, ceftriaxone, ceftazidime) available at the POSGH. The Hospital Pharmacy identified warded patients from the respective services based on the prescriptions dispensed. Patient characteristics, clinical data and laboratory investigations were obtained from the hospital records. While we did consider the course of antibiotic duration in defining appropriateness of therapy, we did not analyse data based on use per patient days because these data were not clearly available from the records. Specific data on the category of service, concomitant disease and drug therapy, organ system with infection, invasive/indwelling devices and the 3GC used were collected using a standardized instrument.

### Case definition of infection

An infection was deemed to be present when the physician's diagnosis of infection was a differential diagnosis stated in the chart and/or in a patient who had a fever (>100.4 C) and elevated WBC Count >12,000/cmm.

On review all patients with diabetes and HIV had a clinical diagnosis of infection stated in the records.

### Criteria for appropriate prescription

The appropriateness of antibiotic therapy was determined using the criteria described in the Sanford Antibiotic Guide, 2000. Therapy was deemed to be inappropriate based on any of the following parameters: the type of therapy (prophylactic and empiric), combination of antibiotics, the route of administration, the dose, and the duration of therapy. The Sanford guide is widely accepted in Trinidad and the Caribbean as a reference for making appropriate therapeutic recommendations and is also used as a reference manual by medical microbiologists. No other guidelines are currently available in Trinidad. The course of therapy was considered a parameter of appropriate therapy, but a separate analysis based on this parameter was not possible since the information was not always available from the patient records.

### Cost of antibiotic treatment

The direct cost of total antibiotic treatment was computed using the data obtained from the National Insurance Property Development Corporation, which is the agency contracted by the Ministry of Health for procurement and distribution of drugs in the public sector health institutes.

### Statistical analysis

Data were analysed using EPI Info 6.4 (CDC, Atlanta GA) and categorical variables were compared using the Odds Ratio (95% CI) and the chi-square (X^2^) tests.

## Results

One hundred and ninety two (192) adult patients admitted to the POSGH were treated with 3GCs during the first six months of 2000, providing a prevalence rate of use of 9.5 patients per 1000 admissions, (192/20146). The rate of use was higher (p < 0.05) in those patients utilizing the surgical and gynecological services (21 per 1000 admissions) compared with those admitted in the medical and orthopedic services (8 per 1000 admissions). One hundred and twenty seven (66%) patients received ceftriaxone, cefotaxime was prescribed for 51 (26.5%) patients, and ceftazdime was administered to 14 (7.5%) patients.

The demographic characteristics of patients who received a 3GC and factors related to infection and treatment in the study population are shown in Table 1. Fifty eight percent (58%) of patients were females. The mean age of patients was 45 ± 20.56 (SD), years. The use of the third generation cephalosporins was highest in patients utilizing the surgical facilities (44%), followed by patients in the medicine (27.5%), gynecology (15%), and orthopedic (12.5%) divisions. Ceftriaxone was the most widely used agent particularly in surgical patients (61, 48%) compared with those admitted to the medicine 24 (19%), gynecology 17(13%)and orthopedic wards 13(10%). In at least one third of patients (63, 32.8%) factors, which could predispose to infection, were identified, diabetes mellitus and HIV/AIDS were diagnosed in 24.5% and 4.7% of patients respectively.

There were 107(56%) patients who had fever and a high WBC count with leucocytosis and who were considered to have a clinically suspected infection. Thirty six percent of patients who had no clinical evidence of infection had received treatment with a 3GC. The most common site of suspected infection (20.3%) was the skin and soft tissue followed by the respiratory (10.9 %), gastrointestinal (10.7%), and urinary (9.3%) tracts.

Sixty eight percent (131) of patients in this study received empirical antibiotic treatment and in 29.7% of patients these antibiotics were prescribed as prophylactic therapy. Only 4% of patients received 3GCs based on recommended therapeutic regimens. More patients (87%) who were admitted to the medical services received empirical antibiotic treatment compared with patients in the surgical services (68%), (p < 0.05). Prophylactic antibiotic regimens were most frequently prescribed in the orthopedic (50%) and gynecological services (45%) in contrast to patients in surgery (31%) who received 3GCs as prophylactic therapy. However patients in the surgical units (61%) were more likely to be administered two or more antibiotics compared with those receiving medical care in the orthopedic (33%) or gynecological (45%) services (p < 0.01). Single antibiotic therapy was prevalent in the medical, orthopedic, and gynecological services (66%, 55%, and 55% respectively). Seventy-eight percent of those patients who did not display any clinical evidence of infection were treated with one antibiotic. Patients with infections of the skin and soft tissue (75%) and the urinary tract (61%) were more likely to receive treatment with two or more antibiotics compared with patients admitted with respiratory tract infections (48%) (p < 0.05).

Biological samples were not sent to the laboratory in as many as 73% of patients to determine a bacteriological etiology of suspected infection. (Table 2). Of the 52 (27%) patients who were submitted to laboratory investigation, a bacteriological etiology could be established in 25 (48 %) patients. The organisms which were isolated were; *Pseudomonas spp *(7), *Acinetobacter spp (3)*, *Enterobacter spp (2)*, *Klebsiella spp (2)*, *Proteus (2) S. aureus (3)*, and others (8).

An analysis of factors that were associated with the inappropriate use of 3GCs in the patient sample is shown in Table 3. There was an independent association between the type of service and inappropriate use of third generation cephalosporins and the odds of inappropriate therapy with these agents was six times more for patients in the gynecology services (odds ratio 6.58, p < 0.003) compared with patients utilizing the orthopedics (odds ratio, 2.47) and medical (odds ratio 1.80)hospital services More patients received inappropriate antibiotic treatment when the site of infection was not determined (odds ratio 5.05) compared with those in whom the site of infection was located to the skin and soft tissue infection (odds ratio 3.10), and the respiratory tract (odds ratio 2.74). The 3GCs were significantly associated with inappropriate use (odds ratio. 3.50 CI 1.73–7.11) when used as single therapy than when used with multiple antibiotics.

The average duration of patient stay in the hospital was 14 ± 22 days (range 1 day -150 days). Patients in the orthopedic wards were hospitalized for more days (24.5 ± SD 35.28) compared with the number of days patients spent in the medical (12.45), surgical (13.7) and gynecology (7 days) services. Interestingly, the mean duration of stay of patients in whom antibiotic treatment was appropriate, was not significantly different from the number of days that patients who were treated inappropriately were warded. The overall direct costs from use of these antibiotics in this study was TT$ 117,432 (US$ 19251.00). The cost of treatment with these antibiotics in patients who were inappropriately treated was (TT $79,487.00, US$ 13, 30.00) and was twice as high as the cost of treatment for those patients who were treated appropriately (TT$ 35, 138.00, US$ 5767)

## Discussion

The extensive use of third generation cephalosporin antibiotics has caused the emergence of extended spectrum beta-lactamases in Gram-negative bacteria worldwide [3-6]. More third generation cephalosporins are being widely used in hospitals for empirical and prophylactic therapy, and as their use extends across the board, more organisms will develop resistance to them presenting the threat of antimicrobial ineffectiveness in life threatening infections. Several investigators have developed and evaluated cost effective programs and adherence to hospital antibiotic guidelines to control antibiotic abuse [15-17]. The active promotion of guidelines increased appropriate prescribing of 3GCs from 21–52% over three years [18]. Though an Infection Control Committee at the POSGH had developed an antibiotic policy earlier for rational use of antimicrobial agents, the Committee did not formally approve the guidelines, they were not disseminated to all physicians and several were unaware of their formulation. A systematic review and evaluation of the guidelines was never conducted despite increasing antimicrobial resistance to third generation cephalosporins in this hospital [14]. It was therefore believed to be timely to undertake a pharmacy audit of use and cost of third generation cephalosporins in this teaching hospital.

In our study, the prevalence rate of use of 3GCs was lower than the reported rate of 43 per 1000 admissions for ceftriaxone in 51 Victorian Hospitals in Australia of which 82% was prescribed as empirical treatment [19]. Our data reveals extensive overall inappropriate use of these drugs (62%) at the POSGH in Trinidad, with a rate that is higher than an earlier literature report (31%) [20]. Inappropriate use of the cephalosporins was seven times more common in the gynecological services compared with other services (odds ratio 6.58 P < 0.003) in this hospital which provides clinical clerkship teaching for graduating medical students from the University of the West Indies. Inappropriate antibiotic use has been reported from teaching hospitals in Aberdeen with significant empirical overuse [21] in New York [22] in the surgical practice [74%], in China [23] where inappropriate 3GC use was an independent risk factor for significant high mortality, in Malaysia [24] for patients in the medical wards (22–65%), in South Africa [25] for patients in the gynaecology ward (54%), and in Thailand (91%) for all departments [26]. There were twice as many patients who received inappropriate prophylactic or empirical therapy with the 3GCs compared with those who received appropriate treatment. We found higher inappropriate use of cefotaxime and ceftriaxone when these agents were used as single agents rather than in combination therapy with other antimicrobial agents. We believe this practice may have followed from the convenience of a single daily dose by intramuscular injection particularly for ceftriaxone, which was the most frequently used 3GC.

Third generation cephalosporins were introduced in the Caribbean between the late eighties and the early nineties and soon after in 1993, the first isolation of resistant Gram-negative bacilli resistant to them was demonstrated in Barbados [12]. In Jamaica, K. pneumoniae isolates confirmed to be ESBL producers have recently been reported in the University Hospital of the West Indies [13]. The 3GCs were introduced to the POSGH formulary in 1990 and their use increased by 48% and 22% from 1995 to1998 and 1998 to 2001 respectively. Cefotaxime was then, the most widely used member of the class and the only member to be available in the public sector health institutes. Resistance to cefotaxime and ceftriaxone in Trinidad, has been demonstrated for isolates of enterobacter (35%,15%), proteus (28%, 8%), acinetobacter (75%, 61%), providencia (75%, 50%) and klebsiella (6%,5%) for all specimens between January to June 2001 at the Port of Spain General Hospital (unpublished data), but blood culture isolates such as klebsiella (25%) and pseudomonas (90%) were resistant to cefotaxime and/or ceftriaxone. The resistance rate of *Enterobacter spp *to cefotaxime and ceftriaxone was 53% and 37% respectively in the Intensive Care Unit at this hospital. It is interesting that only 4% of prescriptions were based on therapeutic regimen following culture and sensitivity results in our study, and in 73% of patients with suspected infection bacteriological confirmatory tests were never done and 3GCs were prescribed. We believe high inappropriate use of these antibiotics has contributed to increasing multiple antibiotic resistance and extended spectrum beta-lactamase producing enterobacteriaceae observed in this hospital [14].

The cost of inappropriate antibiotic use was twice as much for patients who were treated appropriately and highlights irrational antibiotic consumption at the hospital, prompting protocols for rational antibiotic prescribing and utilization review. The World Health Organization has recommended multifaceted strategies to improve hospital-prescribing practices such as the development of consensus guidelines, educational activities and rapid feedback to prescribers about inappropriate use to reduce overuse of antibiotics in the hospital [27]. Restriction of cephalosporin use has demonstrated significant cost savings and improved antibiotic susceptibility with reduced infection-related hospital mortality in critically ill patients [28]. Restructuring the formulary to rationalize antimicrobial use with restricted use of third generation cephalosporins would impact positively in curtailing antibiotic resistance and decrease selective pressure from the overuse of these agents. Moreover the spiraling costs of unwanted antibiotic therapy will be limited with resultant benefits for the consumer and the health care provider in the public health sector. The study highlights the need for an antibiotic audit and invites an ongoing peer audit.

## Conclusions

This first audit on 3GC use from the Caribbean demonstrates inappropriate use of these drugs in tertiary care and presents opportunities to develop consensus guidelines for rational use of these drugs in hospitals. We recommend microbiological services in the hospitals undertake continuous surveillance of resistance patterns, to guide in the development of prescribing guidelines. Such guidelines should be widely disseminated and implemented in these institutions, so that prescribers remain informed of rational therapeutics. This study highlights the need to constitute an antibiotic monitoring team comprising a pharmacist, physician, medical microbiologist and infection control nurse to periodically review and evaluate the use and cost of antibiotics at the major tertiary care hospital in Trinidad, to assist clinicians in optimizing clinical care of patients with Gram-negative infections.

## List of abbreviations

3GCs Third generation cephaosporins

ESBLs Extended spectrum Beta lactamses

POSGH General Hospital, Port of Spain

## Competing interests

The author(s) declare that they have no competing interests.

## Authors' contributions

LMPP conceived the study, the design, co-ordinated it and prepared the final manuscript. MP, HR and KT did the data collection. PP assisted with the co-ordination, did the analysis and prepared the draft manuscript. All authors saw the final manuscript and made contributions.

## Pre-publication history

The pre-publication history for this paper can be accessed here:



## Figures and Tables

**Table 1:** Characteristics of patients treated with 3^rd ^generation cephalosporins at the POSGH

**Characteristics**	**No (%)**
**Gender**	
Male	84 (42)
Female	111(58)
**Age: **Mean SD (Range)	45 ± 20.56(13–90 years)
**Category of service**	
Surgery	84(43.8)
Medicine	53(27.6)
Gynaecology	29(15.1)
Orthopaedics	24(12.5)
Others (CNS, Genital, Septicaemia)	2
**Predisposing Factors**	
None	129(67.2)
Diabetes Mellitus	47(24.5)
HIV/AIDS	9 (4.7)
**Infection characteristics**	
Clinically suspected	108(56)
Clinically absent	84(44)
Site Unknown	69(35.9)
Skin and Soft tissue	39(20.3)
Respiratory tract	21(10.9)
Gastrointestinal tract	20(10.7)
Urinary tract	18(9.3)
Others	25(13.0)
**Antibiotic Use**	
Prophylactic	57 (29.7)
Empiric	131(68.2)
Therapeutic	4(2.0)
Single drug	94(49.6)
Two or more drugs	94(49.6)
Appropriate	62(32.3)
Inappropriate	124(64.6) 6(3.1)
Undetermined	
**Bacteriological Investigations**	
Done	52 (27)
Not done	140 (73)

**Table 2:** Microbiology investigations in patients (192) prescribed third generation cephalosporins

**Type of Service**	**No.**	**Investigations Not done (%)**	**Investigations Done (%)**	**Positives (n = 52) (%)**
Medicine	53	30 (55.7)	23 (44.3)	9 (39.0)
Surgery	84	69 (81.1)	15 (18.9)	10(66.6)
Gynecology	29	23 (79.3)	6 (20.7)	2 (33.3)
Orthopedics	24	16 (66.6)	8 (33.4)	3 (37.5)
Others	2	2		
Total	192	140 (73)	52 (27)	25(48.0)

**Table 3:** Factors associated with appropriate 3rd generation cephalosporin use at the POSGH, Jan- June 2000.

**Variables**	**Appropriate Use (n = 62)**	**Inappropriate Use (n = 124)**	**Odds ratio (95% CI)**	**p Value**
**Gender**				
Male	25	37		
Female	53	71	0.91 (0.46–1.76)	0.88
**Age **(mean ± SD)	42 (18.73)	46.61(21.63)		NS
**Service**				
Gynecology	3	24	6.58 (1.69–29.89)	p < 0.003
Orthopedics	6	18	2.47 (0.081–7.00)	
Medicine	16	35	1.80 (0.81–4.01)	
Surgery	37	45	1.00	
**Infection**				
Present	44	60	2.61 (1.30–8.28)	p < 0.003
Absent	18	64		
**Type of infection**				
Site unknown	15	53	5.05 (1.45–18.10)	p < 0.007
Skin and soft tissue	14	24	4.29 (0.94–20.71	
Respiratory	6	13	3.13(.0.88–11.35)	
Urinary tract	10	7	1.00	
**Therapy**				
Prophylactic	17	39		
Empiric	45	81	1.27 (0.62–2.65)	p < 0.48
**Multiple therapy**				
Single antibiotic	18	73		
2 or more antibiotics	44	51	3.50(1.73–7.11)	p < 0.001
